# Anthraquinolone and quinolizine derivatives as an alley of future treatment for COVID-19: an in silico machine learning hypothesis

**DOI:** 10.1038/s41598-021-97031-x

**Published:** 2021-09-09

**Authors:** Nikhil Samarth, Ritika Kabra, Shailza Singh

**Affiliations:** National Centre for Cell Science, NCCS Complex, Ganeshkhind, SP Pune University Campus, Pune, 411007 India

**Keywords:** Computational biology and bioinformatics, Drug discovery

## Abstract

Coronavirus disease 2019 (Covid-19), caused by novel severe acute respiratory syndrome coronavirus (SARS-CoV-2), has come to the fore in Wuhan, China in December 2019 and has been spreading expeditiously all over the world due to its high transmissibility and pathogenicity. From the outbreak of COVID-19, many efforts are being made to find a way to fight this pandemic. More than 300 clinical trials are ongoing to investigate the potential therapeutic option for preventing/treating COVID-19. Considering the critical role of SARS-CoV-2 main protease (M^pro^) in pathogenesis being primarily involved in polyprotein processing and virus maturation, it makes SARS-CoV-2 main protease (M^pro^) as an attractive and promising antiviral target. Thus, in our study, we focused on SARS-CoV-2 main protease (M^pro^), used machine learning algorithms and virtually screened small derivatives of anthraquinolone and quinolizine from PubChem that may act as potential inhibitor. Prioritisation of cavity atoms obtained through pharmacophore mapping and other physicochemical descriptors of the derivatives helped mapped important chemical features for ligand binding interaction and also for synergistic studies with molecular docking. Subsequently, these studies outcome were supported through simulation trajectories that further proved anthraquinolone and quinolizine derivatives as potential small molecules to be tested experimentally in treating COVID-19 patients.

## Introduction

The upsurge of Coronavirus disease 2019 (COVID-19), caused by the novel Severe Acute Respiratory Syndrome Coronavirus 2 (SARS-CoV-2) possess a severe threat globally. Since 2005, several new coronaviruses have been reported as SARS-CoV and MERS-CoV, many of them have come up from ancestral bat viruses^1^. In late December 2019, hospitals in Wuhan reported crowds of patients presenting symptoms of pneumonia from an unknown cause. The outbreak of this epidemiology was linked to the seafood and wet animal wholesale market in Wuhan, Hubei Province, China. Zhu et al. spotted the source of pneumonia clusters, and described that it is a novel coronavirus which was detected from lower respiratory tract samples of these patients^2^. On January 29th 2020, World Health Organisation declared “2019 nCoV” as a public health emergency for the entire world^1^. Since then, COVID-19 has become pandemic with worsening effects on world public health and economy.

Among the *Coronaviridae, Roniviridae and Arteriviridae* families of order Nidovirales, coronavirus comes from *Coronaviridae* family*.* Almost all coronaviruses have a common morphology with a positive-sense single-stranded RNA genome of up to 31 kb length^3^. Coronaviruses are distinguished by their club-like spike projections on the surface, and has an unusual large RNA genome with a unique strategy of replication. The genome of coronavirus comprises of approximately 30,000 nucleotides encoding four essential structural proteins which are Envelope (E) Membrane (M), Nucleocapsid (N), and Spike (S) proteins, and 16 non-structural proteins from two overlapping open reading frames, ORF1a and ORF1b which arises from replicase polyproteins, pp1ab^4^.

The replicase gene of SARS-CoV-2 encodes for polyproteins, pp1a and pp1ab, which are required for viral replication and transcription. Initially, ORFs 1a and 1b are synthesized into two overlapping polyproteins, which are indistinguishable at the *N*-terminus but, pp1ab has a *C*-terminal extension because of frame-shift mutation. These polyproteins act as precursors of proteins in the transcription–replication complex. As functional polypeptides of the structural proteins (S, M, E and N), replicase and polymerase are released from polyproteins, the proteolytic process becomes very vital. This process is executed by a chymotrypsin-fold proteinase named, the Main protease (M^pro^)^5,6^.

This chymotrypsin-like protease, termed M^pro^ shares some similarities with the 3C proteases of Picornaviruses^3^. It also plays an important role in polyprotein processing and virus maturation, hence, it is considered to be an interesting target for antiviral drug designing as an approach towards treatment of COVID-19. Considering the viewpoint for drug designing, the M^pro^ has been recommended as an enticing drug target due to its significance in the cleavage of the polyprotein into functional polypeptides^7^.

Studies have reported that M^pro^ of all the coronaviruses are highly conserved with respect to their sequences and structures^8^. These features have together contributed in its functional importance, and have made M^pro^ as an attractive target^8,9^. Upon intense screening of various chemical libraries, several small molecules were identified as potent SARS coronavirus protease inhibitors^10–12^.

**Figure 1:**
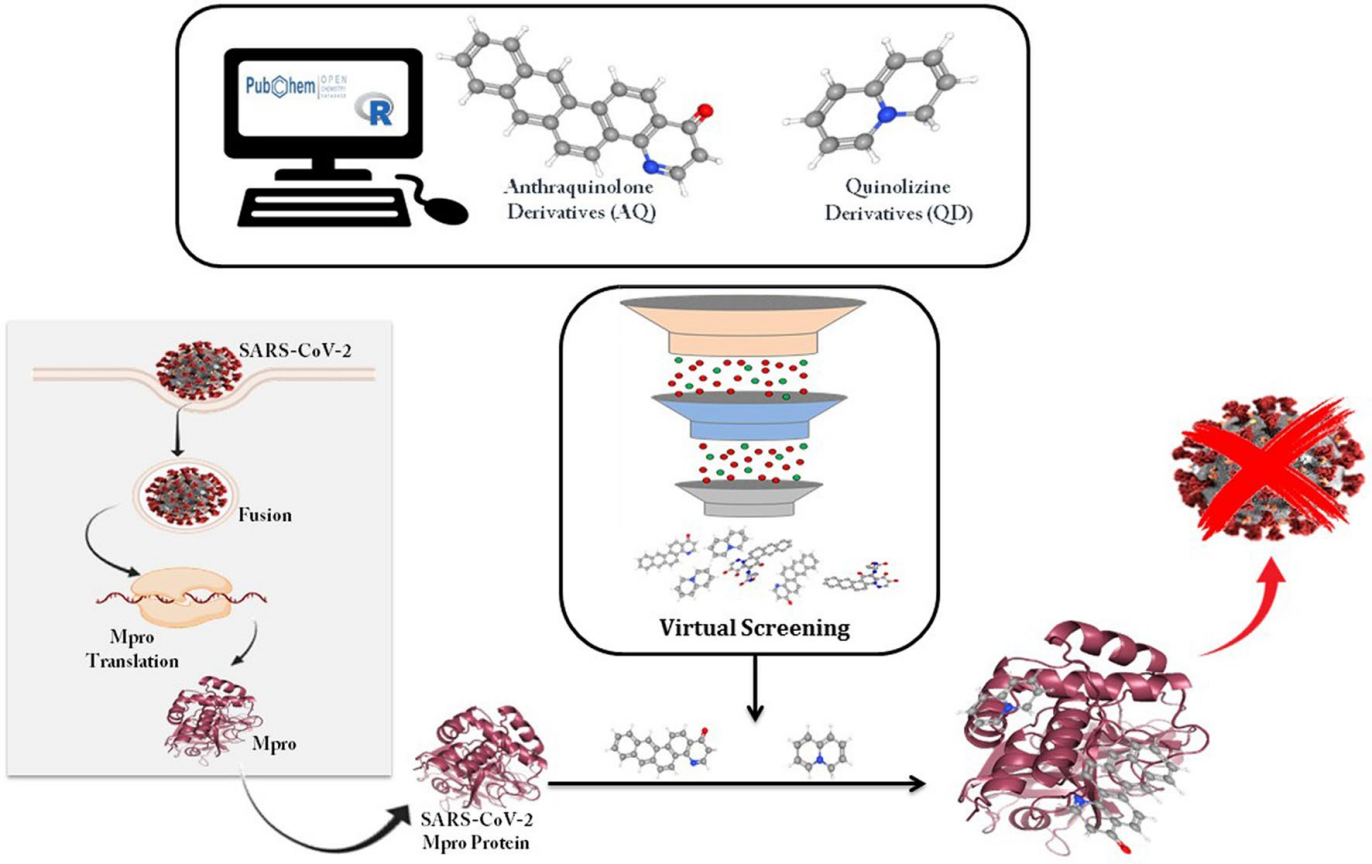
Diagrammatic representation of running machine learning algorithms and Virtual Screening performed for Anthraquinolone (AQ) and Quinolizine (QZ) derivatives against SARS-CoV-2 M^pro^ protease.

Much studies have been reported in the direction of efficacy of antimalarial agents chloroquine and hydroxychloroquine for treating SARS-CoV-2^13–16^. In the current study, we have used Anthraquinolone derivatives (AQ), known to exhibit antimalarial properties^17^ and Quinolizine derivatives (QZ) which are used as repurposed drugs for treating Covid-19^18^. The efficacy of AQ and QZ derivatives as effective inhibitors of SARS-CoV-2 M^pro^ was investigated using cutting-edge computational methods. The outline of the study has been demonstrated in Fig. 1.

## Materials and methods

### Machine learning-based virtual screening

**Figure 2:**
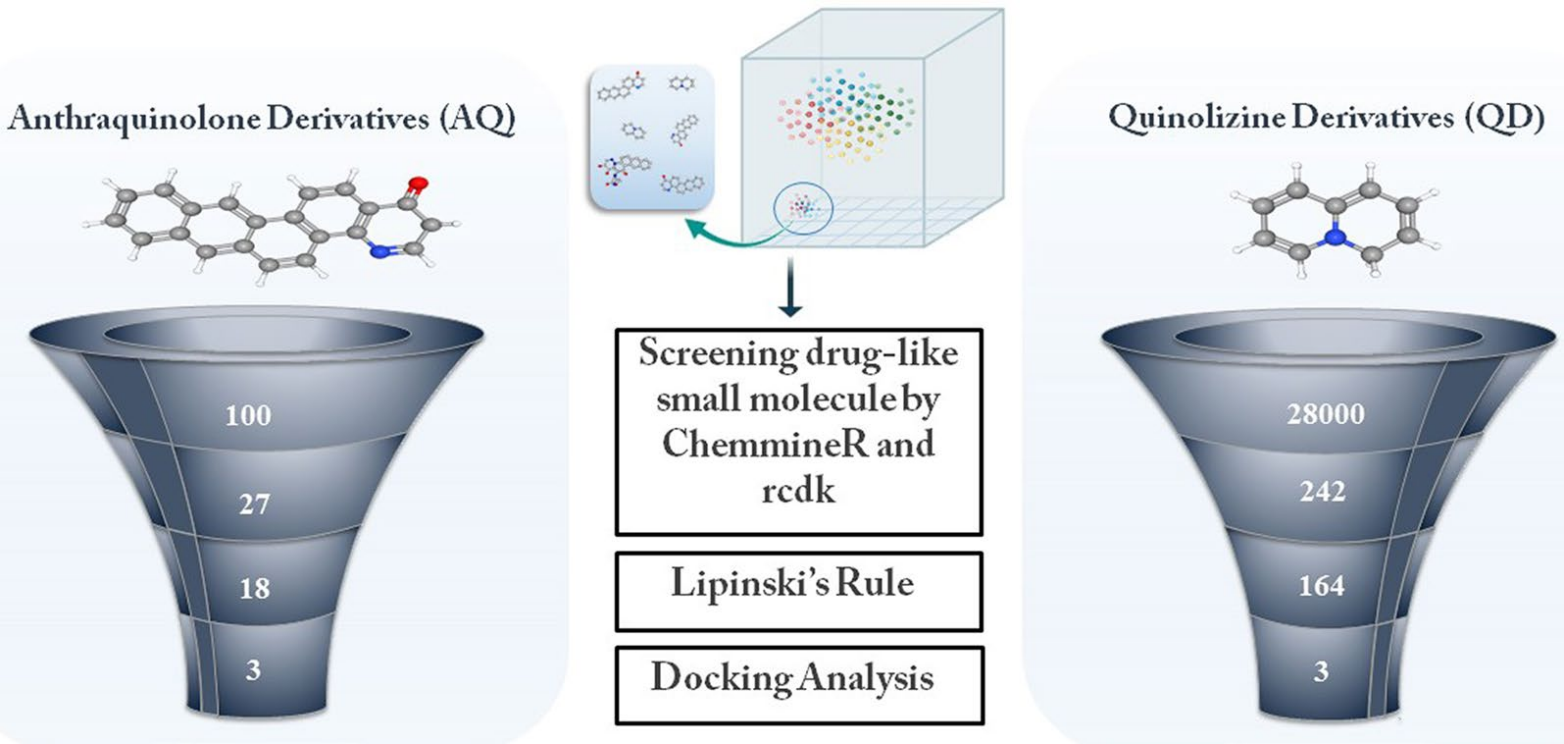
Pipeline of screened AQ and QZ derivatives to top 3 most potent derivatives.

In this study, we have used AQ and QZ derivatives for potential hits by virtual screening. The derivatives of AQ and QZ were screened and downloaded from PubChem^19^, a chemical compound repository consisting of more than 10 million records of compounds for virtual screening. The library consisted of around 28,000 QZ related derivatives and 100 AQ related derivatives. We have used The R Foundation for Statistical Computing,http://www.r-project.org/foundation) with The Comprehensive R Archive Network (CRAN) version 3.5.3 to parse all the available chemical data and implement the machine learning algorithms for association of these datasets. ChemmineR (*CRAN*: *ChemmineR* and Rcdk (*CRAN: Rcdk*) were used to convert the SDF sets and chemical structures were assessed with respect to the stereochemistry, common functional groups, torsions and other salient parameters (R codes provided as supplementary material). Later each of these structural datasets were laid with significant cut-offs and all the compounds were screened and filtered based on similarity and Lipinski's rule^20,21^, a thumb rule to gauge if a chemical compound has a pharmacological or biological activity and also to check if it’s an orally active drug in humans. For screening of these derivatives, 3D structures were downloaded in SDF format from PubChem. These SDF file were then converted to PDB files by OpenBable software^22^, which is mainly used for interconverting chemical file formats. For further refinement, the PDB file of derivatives were uploaded in Lipinski Rule of Five application^23^, which helped in distinguishing between drug-like and non-drug like molecules. Screening process of small derivatives of AQ and QZ has been depicted in Fig. 2.

### Molecular docking

Molecular Docking is a frequently used method in the structure based drug design. It can be used to elucidate the interactions between a protein and a small molecule. Basically, it searches for an appropriate binding of the ligand that energetically and geometrically fits into the protein binding site. Molecular docking enables us to predict the intermolecular framework established between a protein and a small molecule. Further it recommends the binding poses responsible for inhibition of the protein.

We used AutoDockVina tool 1.5.6^24^, a molecular docking software, which provided an accessible interface for processing ligands and targets, polar hydrogen atoms and Gasteiger charges can also be easily added. The crystal structure of SARS-CoV-2 main protease (PDB ID: 6XA4) has been downloaded in PDB format from the Protein Data Bank (RCSB PDB,http://www.rcsb.org/)^25^. After the screening of derivatives of AQ and QZ, the potential derivatives were docked against SARS-CoV-2 M^pro^. The hits of ligand-target complex having least binding energy poses were saved in PDB file format. Further, the interactions of docked files were analysed by LigPlot v.2.2^26^.

### Pharmacophore modeling

**Figure 3:**
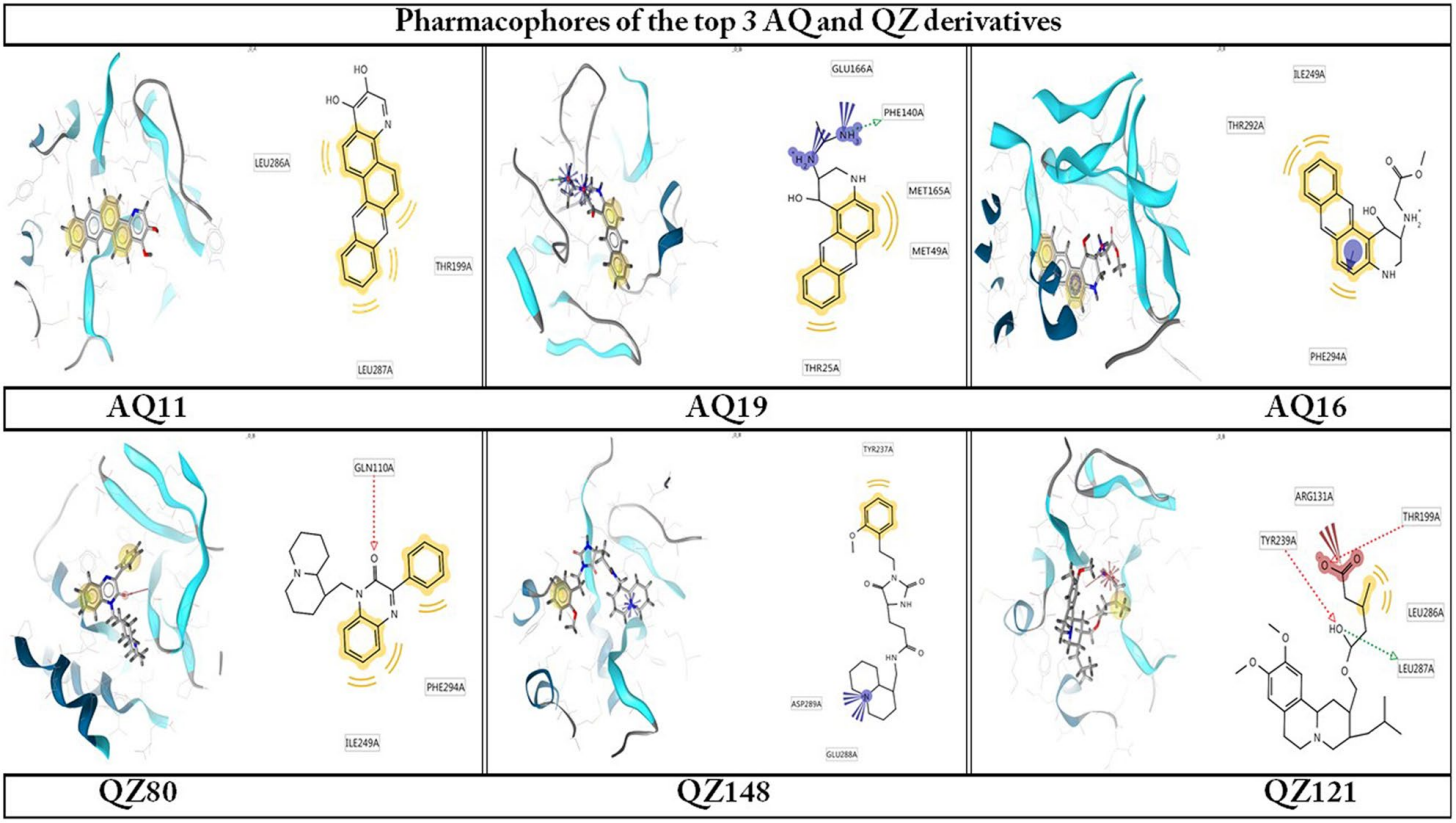
The 3D and 2D structure build pharmacophore models of top 3 AQ and QZ derivatives interacting with Mpro protein(Yellow spheres indicating the hydrophobic interactions, Red coloured arrows indicating H-bond acceptors, Green coloured arrows indicating H-bond and blue stars like indicating the positive ionisable groups.

This technique directly deals with 3D structure of Protein–Ligand complex. It helps to decide the interacting points in between protein and ligand by pinpointing appropriate ligand binding site of the protein^27^. To identify the pharmacophoric features of the top hits of AQ and QZ derivatives obtained (Fig. 3) we have used LigandScout-4.4.5 build 20200714[i1_10], a computational tool which produces structure based pharmacophore models and explains the protein ligand interactions with discrete pharmacophoric features such as hydrophobic regions, hydrophilic regions, hydrogen bond donors, hydrogen bond acceptors etc.^28^.

### Molecular Dynamics Simulation

Molecular Dynamics Simulation is a computationally exhaustive method where we mimic the physiological conditions in which our protein resides, in order to observe the behavioural changes in them. It is based on Newton’s second law of motion which defines that force exerted relies on the mass and acceleration of the atoms. Preliminary state of MDS is to create an initial preparatory state of protein which is followed by introduction to interaction potentials (i.e., energy minimization step) and equilibration of system (NPT/NVT ensembles) finally followed by production MD.

We used MD Simulation to validate stability of AQ/QZ derivatives with M^pro^ protein as well as the interactions maintained between them in the physiological conditions with 300 K temperature, 1 bar pressure and pH 7. The system was surrounded by TIP3P water type enclosed in an orthorhombic box. The thermostat and barostat methods used were Nose–Hoover chain and Martyna-Tobias-Klein respectively with NPT type ensemble. The simulations were run for 50 ns chemical time. Upon completion of MD Simulations, RMSD (Root Mean Square Deviation) and RMSF (Root Mean Square Fluctuation) graphs were generated. All the simulation studies were performed using DESMOND 3.2 with maestro-v11.6 (D.E. Shaw Research)^29^.

## Results and discussion

Developing broad-spectrum inhibitors of M^pro^ is a distinctive strategy against SARS-CoV-2 infection, though; it entirely depends on the availability of a conserved target*.* When screening for a target of a potential inhibitor, all structural proteins such as E, M, N, and S were excluded as they had considerable discrepancy among different CoVs. Consecutively, the RNA-dependent RNA polymerase, RNA helicase, and M^pro^ add up to an attractive drug targets along with some of the non-structural proteins. The pivotal roles played by SARS-CoV-2 M^pro^ in directing viral replication and transcription by processing replicase polyproteins, together with the absence of proximally related cellular homologs, makes M^pro^ as a potential target for antiviral drug designing.

### Screening for the potential AQ and QZ derivatives

A total of 100 derivatives of AQ and 28,000 derivatives of QZ were retrieved from PubChem database. These derivatives were then subjected to screening. A total of 22 derivatives of AQ and 242 derivatives of QZ were screened based on substructure similarity with chloroquine and hydroxychloroquine (enlisted in Supplementary File). In accordance with Lipinski’s Rule of Five, these derivatives were further subjected to screening and from which 4 AQ derivatives and 78 QZ derivatives were ruled out. The remaining 18 AQ and 164 QZ derivatives were then subjected to docking studies.

### Molecular docking of AQ and QZ derivatives

**Figure 4:**
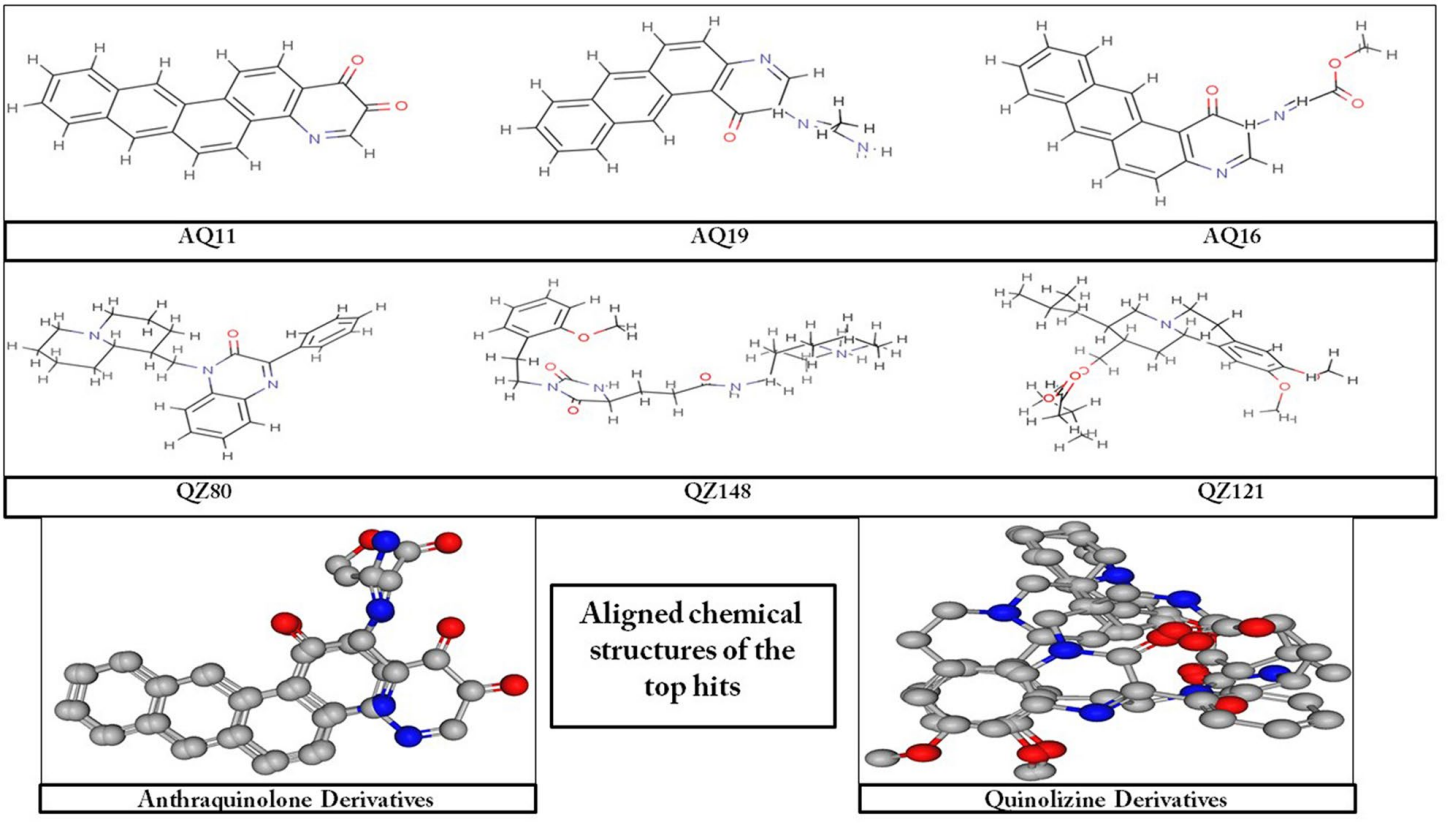
Chemical structures of selected top hits of AQ and QZ.

**Figure 5:**
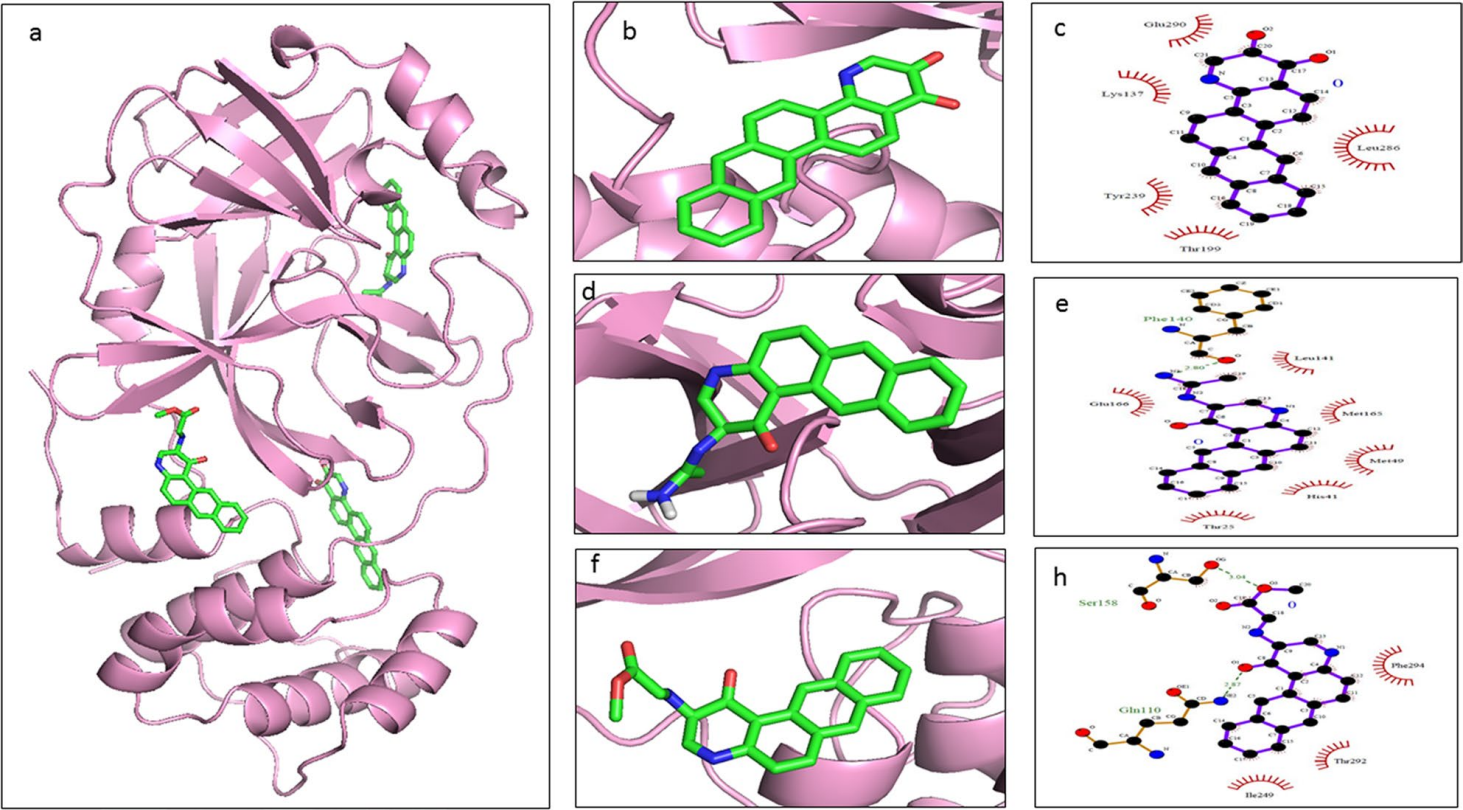
Docked conformations with ligand interacting plots of top 3 AQ derivatives. M^pro^ is represented in pink ribbon format, AQ11 in yellow stick form (**b**), AQ19 in red stick form (**d**) and AQ16 in blue stick form (**f**). (**c**), (**e**) and (**h**) represent the protein–ligand interacting plots for AQ11, AQ19 and AQ16 respectively (Here the green sticks denote Carbon, blue denote Nitrogen and red denote Oxygen atoms of the ligand).

**Figure 6:**
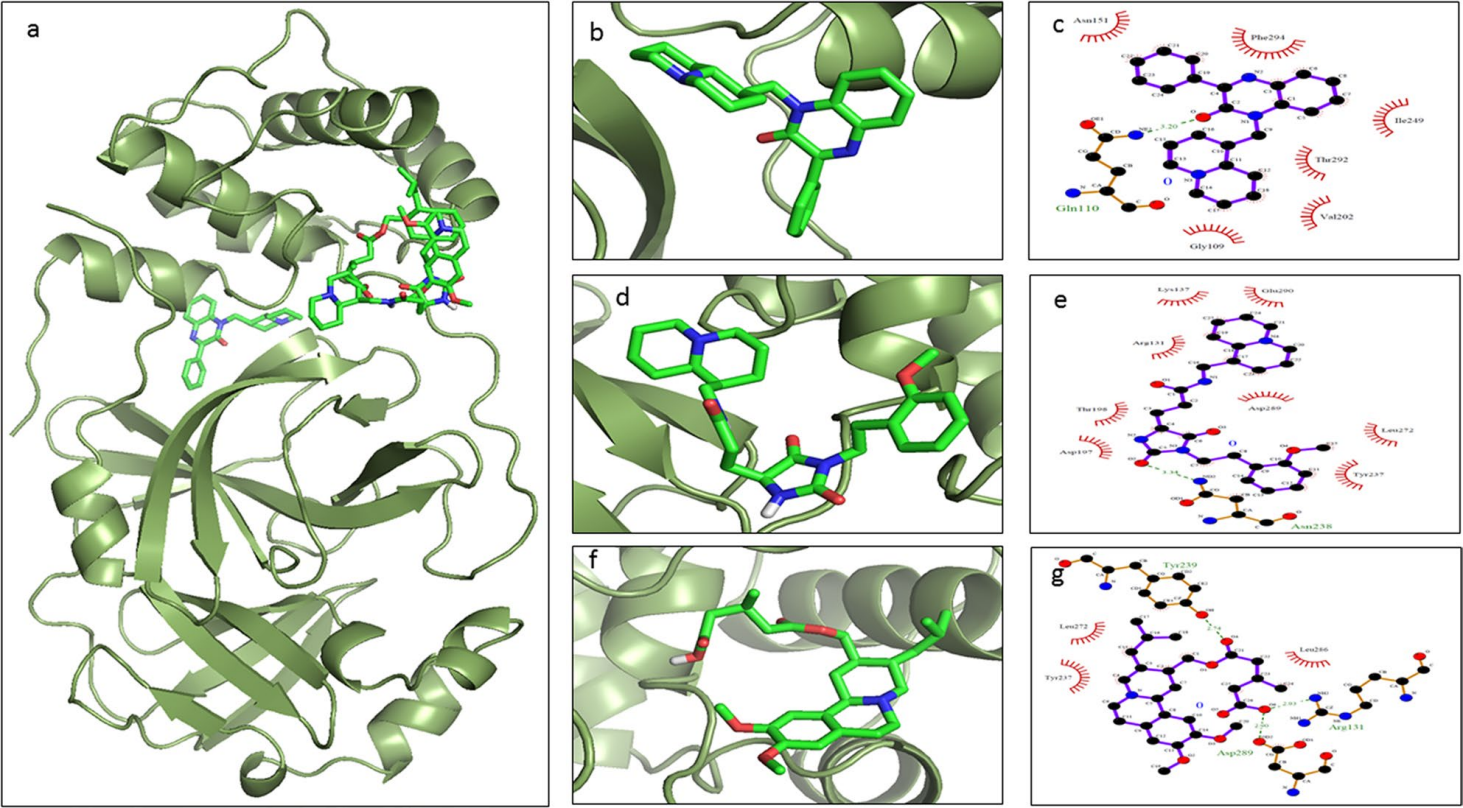
Docked conformations with ligand interacting plots of top 3 QZ derivatives. M^pro^ is represented in green ribbon format, QZ80 in yellow stick form (**b**), QZ148 in red stick form (**d**) and QZ121 in blue stick form (**f**). (**c**), (**e**) and (**h**) represent the protein–ligand interaction plots for QZ80, QZ148 and QZ121 respectively. (Here the green sticks denote Carbon, blue denote Nitrogen and red denote Oxygen atoms of the ligand).

The screened derivatives of AQ and QZ, as mentioned above, were blindly docked with SARS-CoV-2 main protease (M^pro^) (PDB ID: 6XA4). The derivatives of both AQ and QZ gave a good binding affinity. Top hits were sorted out concerning binding energy greater than or equals to − 9.0 kcal/mol. Among these AQ and QZ derivatives, the top three derivatives were selected based on their binding energies. The list of these top hits, along with total interactions and number of H-bond donors and acceptors are shown in Table 1 and the chemical structures are depicted in Fig. 4. The molecular docking studies and their respective interactions with M^pro^ of top hits of AQ derivatives and QZ derivatives are represented in Figs. 5 and 6 respectively. It was observed that Anthrachinolinchinon (AQ11) was having the best affinity for M^pro^ with a binding energy of − 10.1 kcal/mol. Among QZ derivatives, [1-(Octahydro-2H-quinolizin-1-ylmethyl)-3-phenyl-2(1H)-quinoxalinone (QZ80) had a binding energy of − 10.0 kcal/mol.

**Table 1:** Shortlisted AQ and QZ derivatives with interacting residues of M^pro^.

S. no	Derivative	H-bond Donors	H-bond Acceptors	ΔG (kcal/mol)	Interacting residues
1	AQ11	0	3	− 10.1	Lys137, Thr199, Tyr239, Leu286, Glu290
2	AQ19	2	4	− 9.8	Thr25, His41, Met49, Phe140, Leu141, Met165, Glu166
3	AQ16	0	5	− 9.7	Gln110, Ser158, Ile249, Thr292, Phe294
4	QZ80	0	3	− 10.0	Gly109, Gln110, Asn151, Val202, Ile249, Thr292, Phe294
5	QZ148	1	7	− 9.8	Arg131, Lys137, Asp197, Thr198, Tyr237, Asn238, Leu272, Asp289, Glu290
6	QZ121	2	5	− 9.9	Arg131, Try237, Try239, Leu272, Leu286, Asp289

The hydrogen bonds and hydrophobic interactions are important as they stabilize energetically-favoured ligand in an open conformational environment of protein structures and helps in altering binding affinity and drug efficacy. Thus to further analyse the interaction of potential derivatives of both AQ and QZ; they were visualized through LigPlot v.2.2. Anthrachinolinchinon (AQ11) had neither hydrophilic interactions nor H-bonds while the hydrophobic interactions were formed with Lys137, Thr199, Tyr239, Leu286, and Glu290. N-(1-anthrapyridonyl)acetamidine (AQ19) forms H-bond with Phe140 while hydrophobic interactions were observed with Thr25, His41, Met49, Leu141, Met165, and Glu166. The 3rd AQ derivative, methyl N-(1-anthrapyridonyl)iminoacetate (AQ16) interacts with Gln110 and Ser158 forming H-bond while the hydrophobic interactions are formed with Ile249, Thr292 and Phe294. [1-(Octahydro-2H-quinolizin-1-ylmethyl)-3-phenyl-2(1H) quinoxalinone (QZ80) forms a H-bond with Gln110 and has hydrophobic interactions with Gly109, Asn151, Val202, Ile249, Thr292, and Phe294. The 2nd hit of QZ derivative, QZ148 (N- (2,3,4,6,7,8,9,9a-Octahydro-1H-quinolizin-1-ylmethyl)-3-[1-[2-(2-methoxyphenyl)ethyl]-2,5-dioxoimidazolidin-4-yl]propanamide forms H-bond with Asn238 and hydrophobic interactions with Lys5, Tyr126, Lys137, Gly138, Thr199, Tyr237, Tyr239, Leu286, Asp289, and Glu290. [5-[[(2R,3S,11Br)-9,10-dimethoxy-3-(2-methylpropyl)-2,3,4,6,7,11b-hexahydro -1H-benzo[a]quinolizin-2-yl]methoxy]-3-methyl-5-oxopentanoic acid (QZ121, 3rd hit) makes three H-bonds with Arg131, Try239 & Asp289 and hydrophobic interactions with Try237, Leu272, and Leu286. These interacting residues are enlisted in Table 1.

### Molecular Dynamics Simulation of docked complexes

Root mean square deviation (RMSD) is a computational method used to measure quantitative similarity of the atomic co-ordinates between the superimposed structures. It gives the measure of how much a protein confirmation has changed over the course of complete production run (50 ns). Root mean square fluctuation (RMSF) calculates the individual residue flexibility (fluctuation) in contrast to RMSD which calculates the positional differences in the entire structure. RMSF of a protein is plotted against residue number that indicates which amino acid contributes more in the motion of molecule. It was observed that the docked complex structures of AQ11 and AQ16 were stable post 5 ns chemical time with a constant interaction of protein and ligand over the complete simulation time. The AQ11-M^pro^ and AQ16-M^pro^ complexes were stable with RMSD of 1.8 Å and 2.1 Å respectively (Fig. 8). Similar kind of stable conformation was observed in QZ121-M^pro^ complex with RMSD of 2.1 Å (Fig. 9).

**Figure 7:**
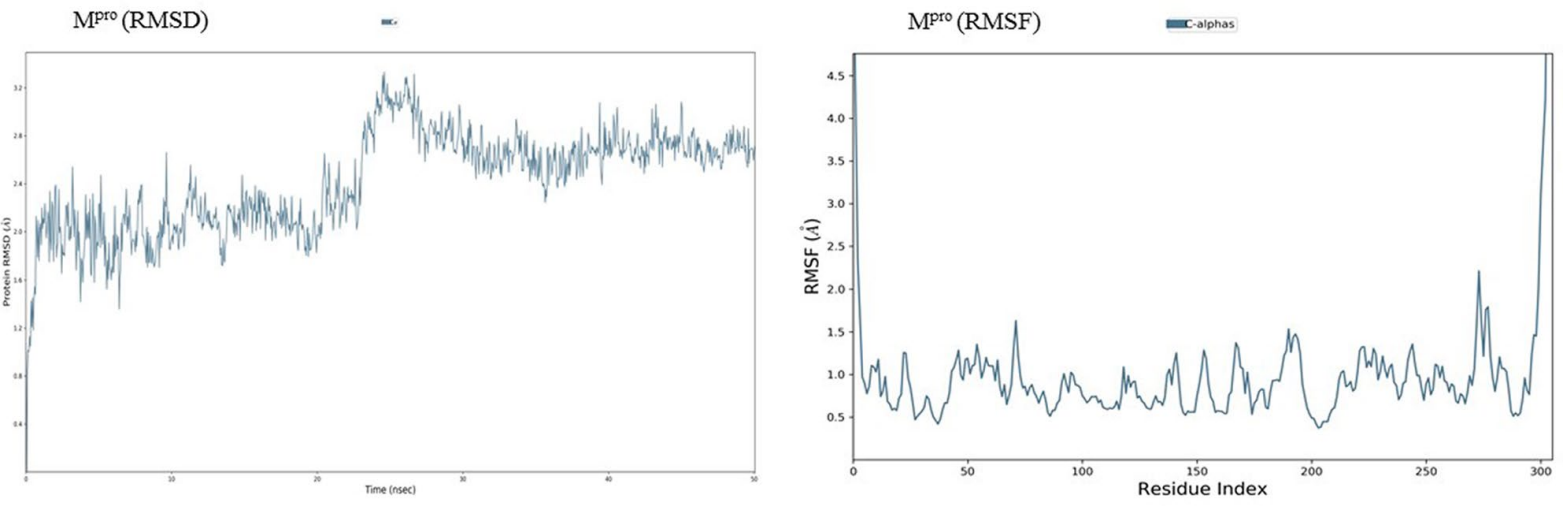
Root mean square deviation (RMSD) and root mean square fluctuation plots of M^pro^.

**Figure 8:**
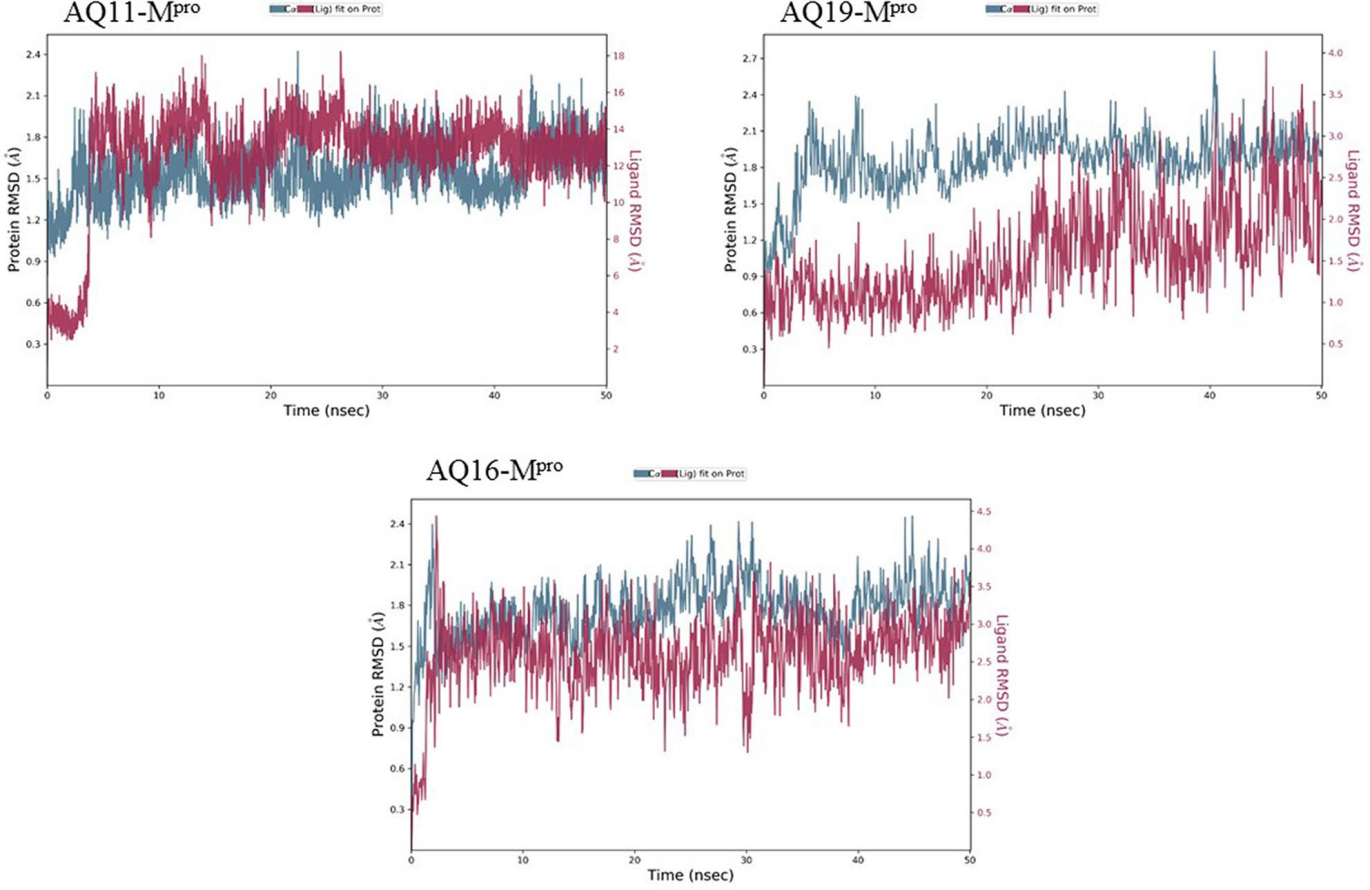
Root mean square deviation (RMSD) plots for docked complexes of AQ-M^pro^.

## Discussion

**Figure 9:**
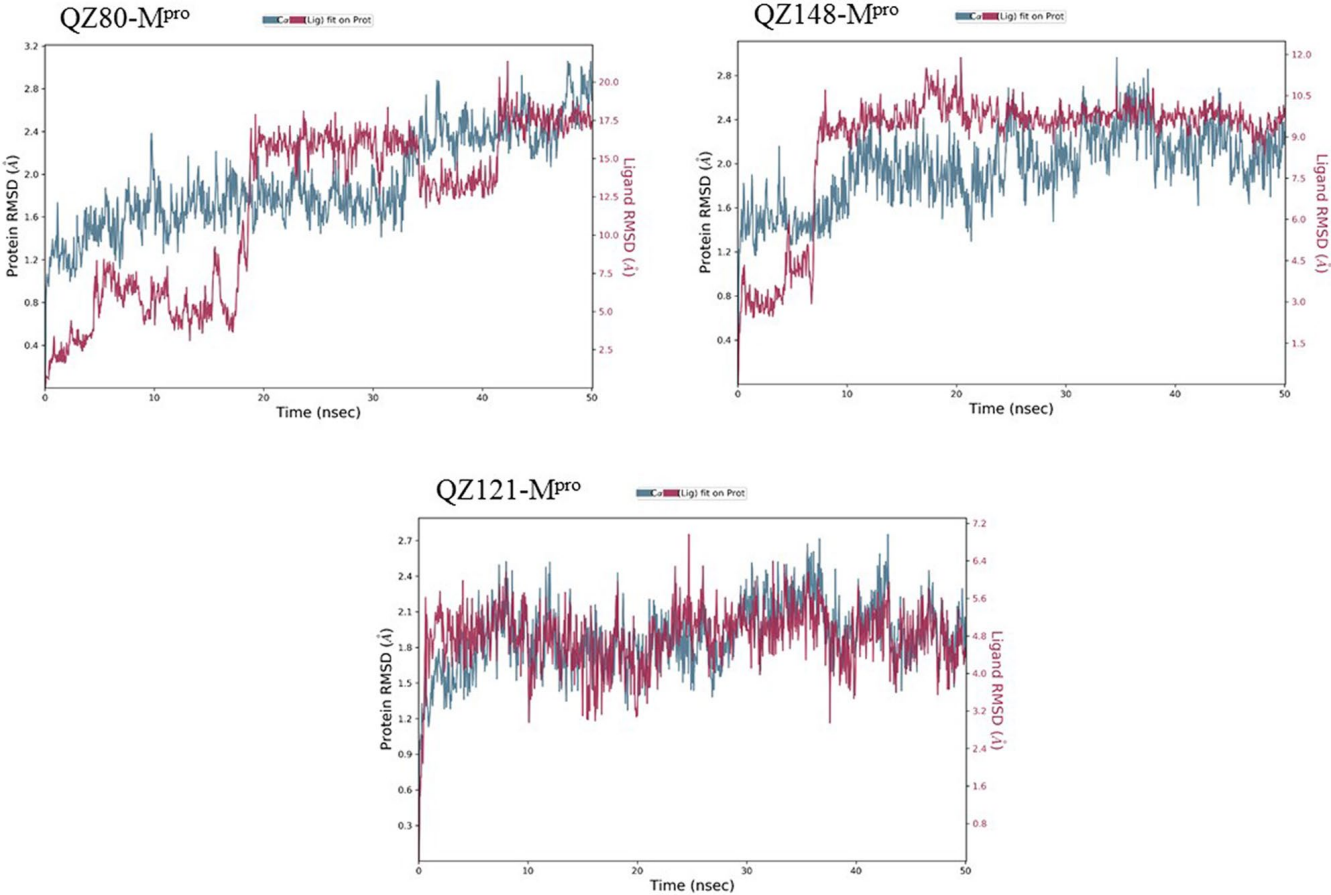
Root mean square deviation (RMSD) plots for docked complexes of QZ-M^pro^.

**Figure 10:**
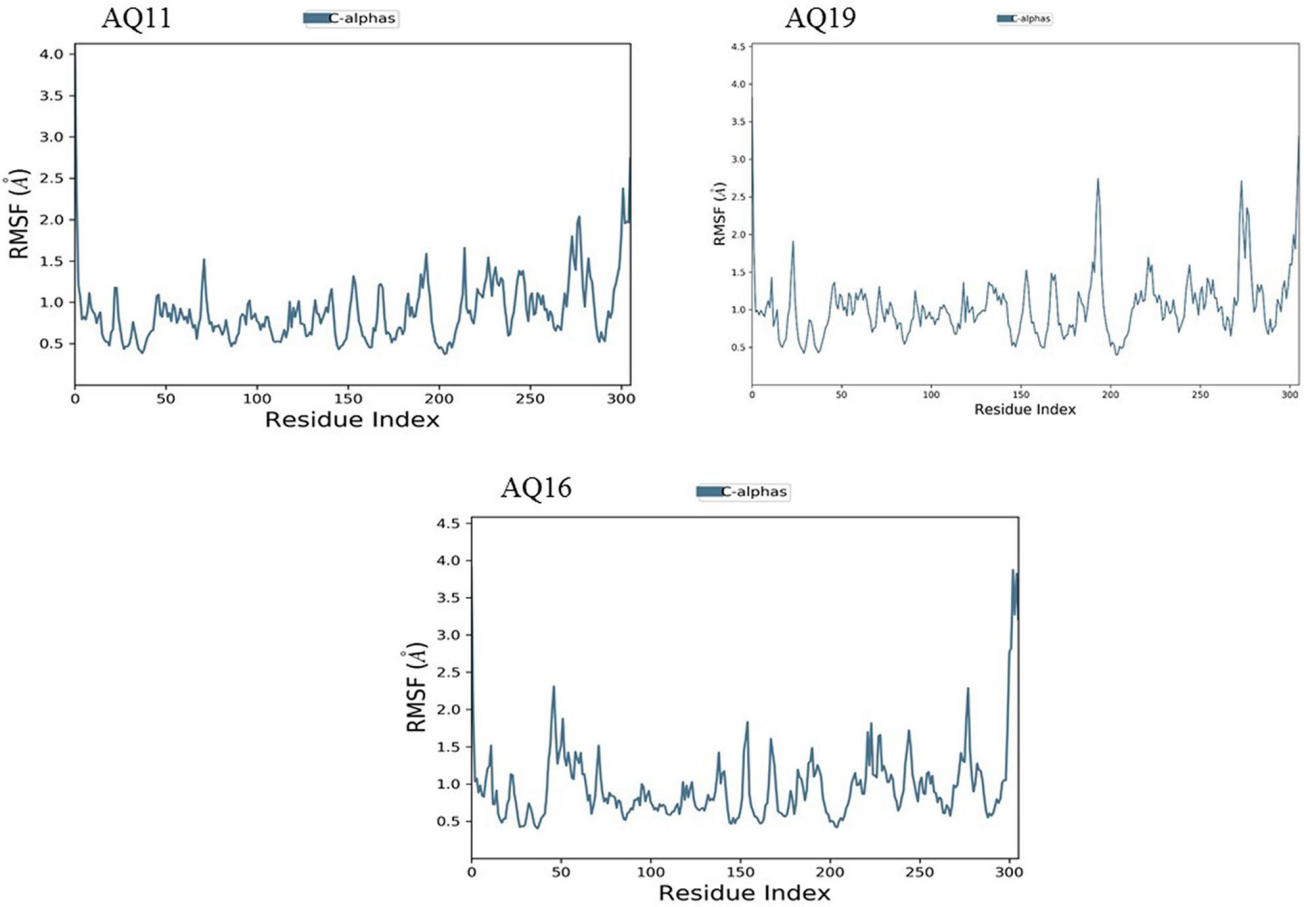
Root mean square fluctuation (RMSF) plots for docked complexes of AQ-M^pro^.

**Figure 11:**
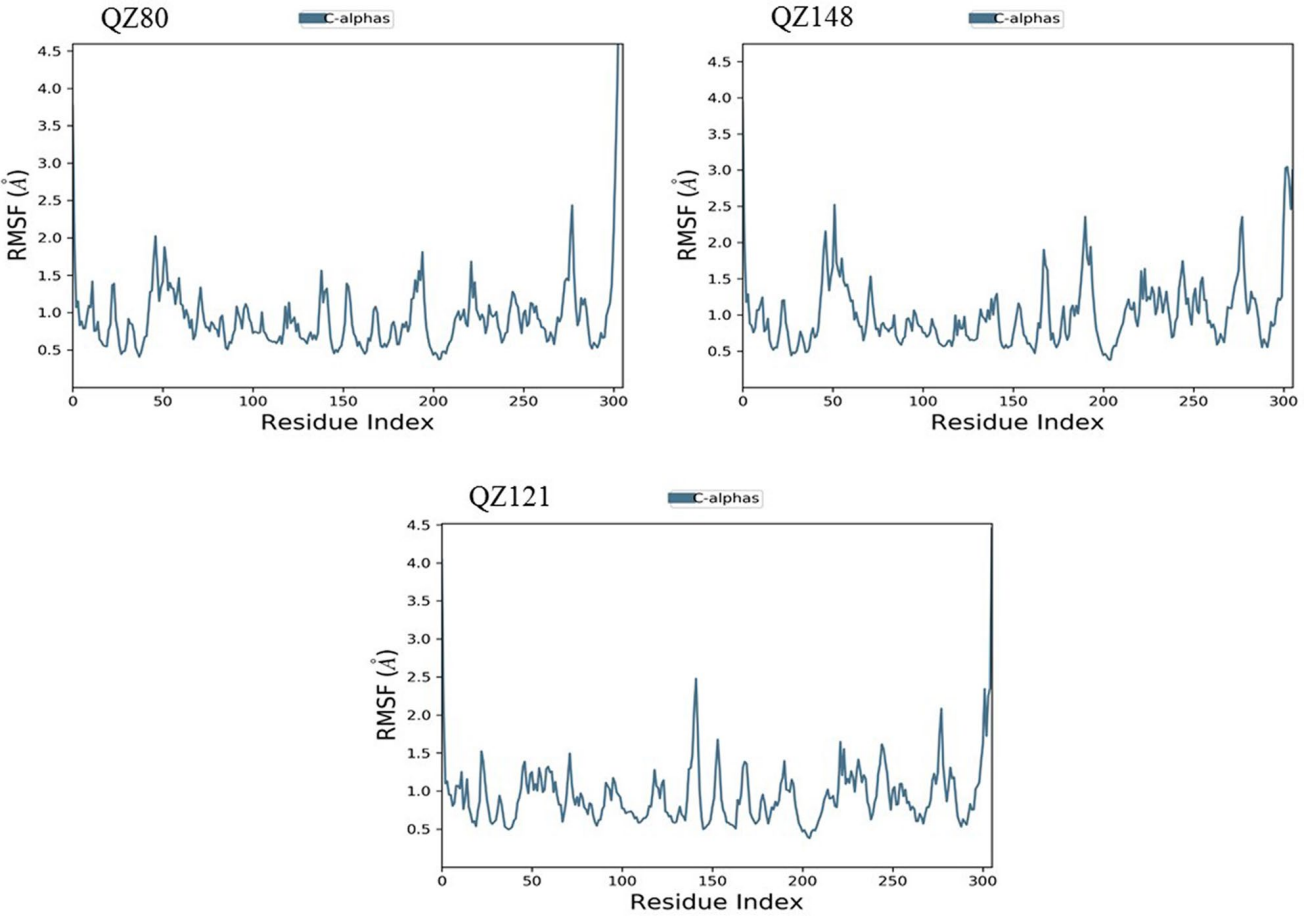
Root mean square fluctuation (RMSF) plots for docked complexes of QZ-M^pro^.

As the invasion of SARS-CoV-2 is extending with a worsening effect on world health due to the unavailability of potential drugs, the need of the hour is to find potential drugs against SARS-CoV-2. In our study, we chose SARS-CoV-2 M^pro^ as a target due to its vital role in replication and transcription of viral proteins. We retrieved derivatives of anthraquinolone and quinolizine (FDA approved drugs used to treat malaria). These derivatives were then screened and the potential ones were subjected to molecular docking analysis to check its credibility of becoming potent inhibitors based on its interaction with SARS-CoV-2 M^pro^. The result showed a good affinity of derivatives towards M^pro^ as depicted by their respective binding energies and interactions. Among derivatives of anthraquinolone, Anthrachinolinchinon(AQ11), N-(1-anthrapyridonyl)acetamidine (AQ19) and methyl N-(1-anthrapyridonyl)iminoacetate (AQ16) and among quinolizine derivatives, [1-(Octahydro-2H-quinolizin-1-ylmethyl)-3-phenyl-2(1H) quinoxalinone (QZ80), QZ148 (N- (2,3,4,6,7,8,9,9a-Octahydro-1H-quinolizin-1-ylmethyl)-3-[1-[2-(2-methoxyphenyl)ethyl]-2,5-dioxoimidazolidin-4-yl]propanamide and [5-[[(2R,3S,11Br)-9,10-dimethoxy-3-(2-methylpropyl)-2,3,4,6,7,11b-hexahydro -1H-benzo[a]quinolizin-2-yl]methoxy]-3-methyl-5-oxopentanoic acid (QZ121) were identified as top hits based on their binding energies. After the Molecular Dynamics Simulation of free M^pro^ and docked complexes AQ-M^pro^, QZ-M^pro^ for 50 ns chemical time, the fluctuations in the structural conformations of free M^pro^ was minimally observed as compared to the docked complexes of AQ11-M^pro^, AQ16-M^pro^ and QZ121-M^pro^ in which the ligand was constantly interacting with the protein (Figs. 7, 8 and 9. According to the RMSF plots of AQ-M^pro^, QZ-M^pro^ and free M^pro^ one can decipher that the secondary structures maintained by the M^pro^ in the free state changes when bound with the ligand suggesting rearranged conformations (Figs. 10 and 11). Based on the above results we could speculate that these derivatives brought about conformational changes in the M^pro^ protein which might hinder the properties of the protein and inhibit the replicability of the virus.

To augment the study, we also did a comparative analysis of our potential hits with known reference compounds such as Ledipasvir, Irbesartan, and Venetoclax which are FDA and NIH approved drugs. These drugs have been docked against M^pro^ by DockCoV2, a drug database for SARS-CoV-2, which calculates binding affinity of the interactions and they have highest binding affinity as compared to the other drugs in the database^30^. After an insightful comparison, data suggested that the binding affinity of our top hits was close to the above mentioned reference compounds, where Ledipasvir, Irbesartan and Venetoclax had binding affinity of − 10.2 kcal/mol, − 9.8 kcal/mol, − 9.7 kcal/mol respectively. As per Lipinski’s rule of five, our top hits satisfies the parameters by falling in the molecular weight range of less than 500 Daltons whereas the reference compounds had higher molecular weight which doesn’t abide to the parameters. The adopted strategy encompasses to predict long term outcomes in terms of sorting the most potent ones, given the cost of clinical trials. We foresee that this *in-silico* study can be substantiated with the *in-vitro* and *in-vivo* analysis for making potential drugs /inhibitor of SARS-CoV-2 M^pro^.

## Supplementary Information

Supplementary Information 1.

Supplementary Information 2.

## Abbreviations

AQ: Anthraquinolone
M^pro^: Main protease
MERS: Middle East Respiratory Syndrome
ORF: Open reading frame
QZ: Quinolizine
SARS-CoV-2: Severe Acute Respiratory Syndrome-Coronavirus 2
