# Supplementary material for: Anthraquinolone and quinolizine derivatives as an alley of future treatment for COVID-19: an in silico machine learning hypothesis
Source: Sci Rep. 2021 Sep 9;11:17915. doi: 10.1038/s41598-021-97031-x (PMC8429452; doi:10.1038/s41598-021-97031-x)
Supplement: Supplementary file 1 — Supplementary Information 1. [file 41598_2021_97031_MOESM1_ESM.doc]

**R codes used to generate ML models for derivatives from PubChem (Structure similarity searching and clustering-an example run)**

BiocManager::install("ChemmineR")

setwd("G:/Compound search/Compounds")

getwd()

data(Compounds)

sdfset <- read.SDFset("G:/Compound search/Compounds/AQ3d.sdf")

header(sdfset[1:376])

header(sdfset[[1]])

atomblock(sdfset[1:376])

atomblock(sdfset[[1]])[1:40,]

bondblock(sdfset[1:376])

datablock(sdfset[1:376])

cid(sdfset)[1:376]

sdfid(sdfset)[1:376]

unique_ids <- makeUnique(sdfid(sdfset))

cid(sdfset) <- unique_ids

blockmatrix <- datablock2ma(datablocklist=datablock(sdfset))

numchar <- splitNumChar(blockmatrix=blockmatrix)

propma <- data.frame(MF=MF(sdfset), MW=MW(sdfset), atomcountMA(sdfset))

propma[1:376, ]

datablock(sdfset) <- propma

datablock(sdfset[1])

grepSDFset("87990657", sdfset, field="datablock", mode="subset")

grepSDFset("87990657", sdfset, field="datablock", mode="index")

write.SDF(sdfset[1:10], file="sub.sdf", sig=TRUE)

write.SDF(sdfset[1:376], file="sub1.sdf", sig=TRUE)

plot(sdfset[1:10], print=FALSE)

plot(sdfset[1:20], print=FALSE)

plot(sdfset[1:50], print=FALSE)

plot(sdfset[1:30], print=FALSE)

plot(sdfset[1:40], print=FALSE)

plot(sdfset[1:4], print=FALSE)

sdf.visualize(sdfset[1:4])

sdf.visualize(sdfset[1:376])

apset <- sdf2ap(sdfset)

propma <- atomcountMA(sdfset, addH=FALSE)

boxplot(propma, main="Atom Frequency")

boxplot(rowSums(propma), main="All Atom Frequency")

propma <- data.frame(MF=MF(sdfset, addH=FALSE), MW=MW(sdfset, addH=FALSE),

Ncharges=sapply(bonds(sdfset, type="charge"), length),

atomcountMA(sdfset, addH=FALSE),

groups(sdfset, type="countMA"),

rings(sdfset, upper=6, type="count", arom=TRUE))

propma[1:376,]

BiocManager::install("ChemmineOB")

library(ChemmineOB)

propOB(sdfset[1:376])

apset <- sdf2ap(sdfset)

fpset <- desc2fp(apset, descnames=1024, type="FPset")

fpset

fpchar <- desc2fp(x=apset, descnames=1024, type="character")

fpchar <- as.character(fpset)

fpma <- as.matrix(fpset)

fpset <- as(fpma, "FPset")

fpSim(x=fpset[1], y=fpset, method="Tanimoto")

simMAap <- sapply(cid(fpset), function(x) fpSim(fpset[x], fpset, sorted=FALSE))

hc <- hclust(as.dist(1-simMAap), method="single")

plot(as.dendrogram(hc), edgePar=list(col=4, lwd=2), horiz=TRUE)

data(pubchemFPencoding)

fpset

fpSim(fpset[1], fpset[2])

fpSim(fpset["10089395"], fpset, method="Tanimoto", cutoff=0.6, top=6)

plot(sdfset[names(cmp.search(apset, apset[6], type=2, cutoff=4))])

clusters <- cmp.cluster(apset, cutoff = c(0.7, 0.8, 0.9))

clusters[1:376,]

fpset <- desc2fp(apset)

clusters2 <- cmp.cluster(fpset, cutoff=c(0.5, 0.7, 0.9), method="Tanimoto")

clusters2[1:376,]

clusters <- cmp.cluster(db=apset, cutoff = c(0.65, 0.5, 0.3), save.distances="distmat.rda")

load("distmat.rda")

cluster.visualize(apset, clusters, size.cutoff=2, quiet = TRUE)

coord <- cluster.visualize(apset, clusters, size.cutoff=1, dimensions=3, quiet=TRUE)

library(scatterplot3d)

scatterplot3d(coord)

library(rcdk)

setwd("G:/Compound search/Compounds")

getwd()

data(AQ3d)

data(Compounds)

mols <- load.molecules( c('AQ3d.sdf') )

iter <- iload.molecules('AQ3d.sdf', type='sdf')

write.molecules(mols, filename='AQ3d.sdf')

view.molecule.2d(mols[[5]], depictor=depictor)

smiles <- c('CCC', 'c1ccccc1', 'CCCC(C)(C)CC(=O)NC')

mols <- parse.smiles(smiles)

get.smiles(mols[[1]])

smiles <- c('CCC', 'CCN', 'CCN(C)(C)',

'c1ccccc1Cc1ccccc1',

'C1CCC1CC(CN(C)(C))CC(=O)CC')

mols <- parse.smiles(smiles)

view.molecule.2d(mols[[1]])

view.molecule.2d(mols)

depictor <- get.depictor(style='cob', abbr='reagents', width=300, height=300)

view.molecule.2d(mols[[5]], depictor=depictor)

mol <- nextElem(iter)

iter <- iload.molecules('AQ3d.sdf', type='sdf')

while(hasNext(iter)) {

mol <- nextElem(iter)

print(get.property(mol, "cdk:Title"))

}

mols <- parse.smiles(c('CC(C)(C)C','c1ccc(Cl)cc1C(=O)O', 'CCC(N)(N)CC'))

query <- '[#11609517]'

matches(query, mols)

dc <- get.desc.categories()

dc

dn <- get.desc.names(dc[4])

dn

aDesc <- eval.desc(mols, dn[4])

allDescs <- eval.desc(mols, dn)

descNames <- unique(unlist(sapply(get.desc.categories(), get.desc.names)))

data(bpdata)

mols <- parse.smiles(bpdata[,1])

descNames <- c(

'org.openscience.cdk.qsar.descriptors.molecular.KierHallSmartsDescriptor',

'org.openscience.cdk.qsar.descriptors.molecular.APolDescriptor',

'org.openscience.cdk.qsar.descriptors.molecular.HBondDonorCountDescriptor')

descs <- eval.desc(mols, descNames)

class(descs)

dim(descs)

model <- lm(BP ~ khs.sCH3 + khs.sF + apol + nHBDon, data.frame(bpdata, descs))

summary(model)

mol <- parse.smiles('CC(=O)CC(=O)NCN')[[1]]

convert.implicit.to.explicit(mols)

get.tpsa(mols)

plot(bpdata$BP, predict(model, descs),

xlab="Observed BP", ylab="Predicted BP",

pch=19, xlim=c(100, 700), ylim=c(100, 700))

abline(0,1, col='red')

data(bpdata)

mols <- parse.smiles(bpdata[,1])

fps <- lapply(mols, get.fingerprint, type='circular')

fp.sim <- fingerprint::fp.sim.matrix(fps, method='tanimoto')

fp.dist <- 1 - fp.sim

cls <- hclust(as.dist(fp.dist))

plot(cls, main='A Clustering of the BP dataset', labels=FALSE)
